# Impact of Intraoperative Norepinephrine Support on Living Donor Liver Transplantation Outcomes: A Retrospective Cohort Study of 430 Children

**DOI:** 10.3389/fphar.2020.01254

**Published:** 2020-08-14

**Authors:** Jiang-Long Chen, Yuan-Li Chen, Bo Qi, Zhi-Ying Pan, Ye-Feng Lu, Wei Zhang, Jiao Zhu, Wei-Feng Yu, Jin-chao Song, Yu-Gang Lu

**Affiliations:** ^1^Department of Anesthesiology, Shanghai Pulmonary Hospital, Tongji University School of Medicine, Shanghai, China; ^2^Department of General Surgery, Children’s Hospital, Shanghai Jiao Tong University School of Medicine, Shanghai, China; ^3^Department of Anesthesiology, Renji Hospital, Shanghai Jiao Tong University School of Medicine, Shanghai, China; ^4^Department of Hepatic Surgery, Renji Hospital, Shanghai Jiao Tong University School of Medicine, Shanghai, China; ^5^Department of Biostatistics, School of Public Health, Fudan University, Shanghai, China; ^6^Department of Anesthesiology, Eastern Hepatobiliary Surgical Hospital, Second Military Medical University, Shanghai, China; ^7^Department of Anesthesiology, Shidong Hospital of Shanghai, University of Shanghai for Science and Technology, Shanghai, China

**Keywords:** norepinephrine, living donor liver transplantation, outcomes, end-stage liver disease, retrospective study

## Abstract

Norepinephrine (NE) is often administered during the perioperative period of liver transplantation to address hemodynamic instability and to improve organ perfusion and oxygen supply. However, its role and safety profile have yet to be evaluated in pediatric living donor liver transplantation (LDLT). We hypothesized that intraoperative NE infusion might affect pediatric LDLT outcomes. A retrospective study of 430 pediatric patients (median [interquartile range] age, 7 [6.10] months; 189 [43.9%] female) receiving LDLT between 2014 and 2016 at Renji Hospital was conducted. We evaluated patient survival among recipients who received intraoperative NE infusion (NE group, 85 recipients) and those that did not (non-NE group, 345 recipients). The number of children aged over 24 months and weighing more than 10 kg in NE group was more than that in non-NE group. And children in NE group had longer operative time, longer anhepatic phase time and more fluid infusion. After multivariate regression analysis and propensity score regression adjusting for confounding factors to determine the influence of intraoperative NE infusion on patient survival, the NE group had a 169% more probability of dying. Although there was no difference in mean arterial pressure changes relative to the baseline between the two groups, we did observe increased heart rates in NE group compared with those of the non-NE group at anhepatic phase (P=0.025), neohepatic phase (P=0.012) and operation end phase (P=0.017) of the operation. In conclusion, intraoperative NE infusion was associated with a poorer prognosis for pediatric LDLT recipients. Therefore, we recommend the application of NE during pediatric LDLT should be carefully re-considered.

## Introduction

Living donor liver transplantation (LDLT) was first introduced in China in June 1997 as a life-saving treatment for children with end-stage liver disease (ESLD) due to the consistent shortage of donor organs. Benefiting from the innovation and development of surgical technique, immunosuppression and perioperative management over the past few decades, pediatric LDLT has achieved promising short- and long-term patient and graft survival (Kasahara et al., 2013; Kasahara et al., 2018). Nowadays LDLT has become the mainstream method for pediatric liver transplantation (LT) in Asia (Chen et al., 2013).

One of the most common anesthetic problems during the whole surgical phase of liver transplantation procedure is hypotension (Perilli et al., 2016; Mizota et al., 2017), which is partially induced by ESLD-related pathophysiological changes, such as portal hypertension (Wright and Rikkers, 2005) and cardiorespiratory changes (Raval et al., 2011). Most LT anesthesiologists have tended to use norepinephrine (NE), which is one of the powerful vasoconstrictors released by sympathetic nerve endings and one of the most widely used cardiovascular active drugs in clinical anesthesia, to maintain hemodynamic stability during LT anesthesia. Previous studies have shown that intraoperative application of vasoconstrictors could reduce the occurrence of postreperfusion syndrome (Ryu et al., 2012), improve renal perfusion (Fayed et al., 2013; Sahmeddini et al., 2013) and reduce the frequency of percutaneous interventions and the length of hospital stay (Reddy et al., 2017). However, there is little evidence focusing on the relationship between intraoperative vasoconstrictor infusion and the prognosis of LT, especially LDLT in children.

Improper perioperative NE infusion in pediatric LDLT patients with ESLD is likely to increase cardiac stress, aggravate cardiac injury, and ultimately lead to poor perioperative prognosis. It has been estimated that 40% to 50% of patients with ESLD have some signs of cirrhotic cardiomyopathy (CCM) (Zardi et al., 2010; Carvalho and Kroll, 2019), and the incidence of CCM in infants with ESLD even higher (Desai et al., 2011). CCM is a severe cardiovascular condition characterized by a hyperdynamic circulatory state, altered diastolic relaxation, impaired contractility, and rhythm abnormalities, particularly QT interval prolongation (Desai et al., 2017; Carvalho and Kroll, 2019). It is a condition easily tolerated because of the near-normal cardiac function at rest, manifesting only under conditions of physical or pharmacological stress. It is exactly this characteristic that causes the insufficiency of a more accurate description of this entity in pediatric patients and a pediatric-specific diagnostic plan. Fortunately, attention has been paid to this situation. In addition, the appeal for more pediatric studies in the field of CCM has been made (Fattouh et al., 2016). Basic research findings suggest that the cardiotoxic effects of bile acids and their varied receptor-mediated functions play a crucial role in their involvement in cardiovascular dysfunction in cirrhosis (Voiosu et al., 2017). A very recent study on pediatric CCM indicates that CCM-associated cardiac changes might impact peritransplant care and posttransplant hospitalization time, but were not correlated with patient survival pre-LT or post-LT, and they were reversed after LT (Junge et al., 2018). However, for these patients with underlying CCM, more attention should be paid to the selection and application of cardiovascular active drugs during pediatric LDLT, so as to avoid increasing cardiac stress and aggravating cardiac injury, and to improve outcomes.

NE is an important regulator of myocardial contractility and metabolism (Rona, 1985). As one of the powerful vasoconstrictors released by sympathetic nerve endings and the adrenal medulla, NE is characterized by α-adrenergic agonistic activity in addition to a weak β-adrenergic agonistic activity. It is the first-line agent to maintain end-organ perfusion pressure, especially in critically ill patients with low systemic vascular resistance. Many centers use it to minimize transfusion volume and fluid administration during the pre-anhepatic and anhepatic phase, and to reduce the occurrence of postreperfusion syndrome during the neohepatic phase of LT (Barjaktarevic et al., 2018). Previous studies have shown that NE-induced, β1-mediated increase in myocardial contractility played a crucial role in increasing the cardiac index of septic, postcardiotomy vasodilatory shock (Nygren et al., 2010; Redfors et al., 2011) and LT (Skytte Larsson et al., 2018) patients. Obviously, the administration of exogenous NE is mandatory to support the failing circulation in critically ill patients. However, in contrast to these short-term benefits, prolonged adrenergic stress is detrimental to the cardiovascular system, especially when the cardiovascular system of these pediatric recipients has been under the cardiotoxic effects of bile acids for a long time and some of them has already shown some signs of CCM.

When blood pressure needs to be rapidly restored, NE is a reliable first-line agent. However, doubts about the utilization of NE have never stopped, mainly because of the adverse effects of NE. Experimental studies have shown that NE infusion caused acute myocardial necrosis (Todd et al., 1985) in dogs, impaired splanchnic hemodynamics and oxygenation in pigs (Priebe et al., 1995), and induced an impairment of hepatic macrocirculation and microcirculation in the early phase after porcine LT (Mehrabi et al., 2005). Clinical evidences have suggested that NE administration in patients with community-acquired septic shock was associated with worse outcome (a 3.5 increase in the 28-day mortality risk) (Povoa et al., 2009), using NE in dialysis-required septic acute kidney injury patients was associated with higher 90-day mortality (Chen and Wu, 2018), about 10% of patients with septic shock on NE infusion had serious adverse events leading greatly to increased mortality (Anantasit et al., 2014), and exogenous administration of NE was a major risk factor for broken-heart syndrome (Kido and Guglin, 2017). Nowadays anesthesiologists have been accustomed to using NE to maintain hemodynamic stability during the perioperative period of LT, however, more prospective clinical investigations should be conducted to determine the impact of NE infusion on outcomes of pediatric LDLT.

Scarce literature and empirical evidence led us to study the influence of intraoperative NE infusion on LDLT outcomes. The aims of this retrospective study were to review our experience over the past few years with LDLT for children and to analyze the effect of intraoperative NE infusion on recipients’ survival following pediatric LDLT.

## Patients and Methods

We retrospectively analyzed the medical data for all living donors and recipients, who underwent primary LDLT surgery from January 1, 2014, to December 31, 2016, at Renji Hospital, Shanghai, China. The last follow-up record was up to June 2018.

Patients were enrolled in this cohort if they: (1) age under 12 years; (2) underwent LDLT for End-Stage Liver Disease (ESLD); (3) available and complete clinical records of the patients’ peri-transplantation procedure. Patients were excluded if they: (1) underwent LT for acute liver failure and cancer; (2) underwent combined organ transplantation, heterotopic transplantation and re-transplantation; (3) incomplete clinical and instrumental follow-up.

A total of 430 patients who met the inclusion and the exclusion criteria were divided into two groups (NE group and non-NE group) according to whether infused NE during surgery. The primary outcome considered in the analysis was patient survival among the different studied groups during follow-up.

Perioperative variables included in the analysis were sex, age, body weight (BW), graft-to-recipient weight ratio (GRWR) measurement, diagnosis of primary diseases, Kasai procedure, Pediatric End-Stage Liver Disease (PELD) score, donor/recipient ABO compatibility, preoperative white blood cell (WBC) and hemoglobin (Hb) levels, operative time, duration of anhepatic phase, amount of blood loss, amount of intraoperative fluid, and red blood cell infusion.

Intraoperative hemodynamic parameters including mean arterial pressure (MAP) and heart rate during the four phases (beginning phase, anhepatic phase, neohepatic phase, and ending phase) of transplantation, and the duration and dosage of NE infusion were extracted from the anesthesia note.

Approval for this study was obtained by the Renji Hospital Ethics Committee.

### Statistical Analysis

Continuous variables are described as median (interquartile range, IQR) and categorical variables as frequencies (percentages). A t test or Wilcoxon rank-sum test was used for continuous variables, and chi-square tests or Fisher’s exact tests were used for categorical variables, as appropriate. A P value < 0.05 was considered statistically significant. Survival probabilities were estimated by the Kaplan-Meier method and the log-rank test was applied for comparisons of survival curves. In order to ascertain the influence of intraoperative NE infusion on LDLT recipients’ survival, multivariate regression, and propensity score regression adjustment were conducted to adjust for potential confounding factors. All analyses were performed using the SPSS 25.0 statistical package (IBM, Inc., Chicago, IL).

## Results

This cohort included 430 children, 85 (19.8%) recipients received intraoperative NE infusion (**Table 1**). The proportion of children aged over 24 months and weighing more than 10 kg in NE group was larger than that in non-NE group (NE group vs. non-NE group, 22.4% vs. 7.5%, 23.5% vs. 12.5%, respectively). In addition, a larger proportion of children in NE group were diagnosed with metabolic diseases (NE group vs. non-NE group, 15.3% vs. 6.1%). Meanwhile, intraoperative factors analysis indicated that children in NE group had longer operative time (NE group vs. non-NE group, Median [IQR], 438.5 min [397–497.5] vs. 398 min [360–451]), longer anhepatic phase (NE group vs. non-NE group, Median [IQR], 40 min [35–51] vs. 38 min [32–45]), more fluid infusion (NE group vs. non-NE group, Median [IQR], 1810 ml [1,550–2,400] vs. 1685 ml [1,450–2,110]) and more blood loss (NE group vs. non-NE group, 200–400 ml, 20.0% vs. 11.9%; > 400 ml, 8.2% vs. 2.0%).

**Table 1:** Baseline Characteristics.

Variables	NE group [n = 85 (19.8%)]	Non-NE group [n = 345 (80.2%)]	*P*
**General Factors**			
Sex mismatch Yes, n (%)	45 (52.9)	172 (49.9)	0.610
No, n (%)	40 (47.1)	173 (50.1)	
Age			<0.001
>24 months, n (%)	19 (22.4)	26 (7.5)	
<=24 months, n (%)	66 (77.6)	319 (92.5)	
BW			0.010
>10 kg, n (%)	20 (23.5)	43 (12.5)	
<=10 kg, n (%)	65 (76.5)	302 (87.5)	
GRWR			0.142^†^
>4, n (%)	3 (3.5)	4 (1.2)	
<=4, n (%)	82 (96.5)	341 (98.8)	
Diagnoses			0.020^†^
Biliary atresia, n (%)	71 (83.5)	320 (92.7)	
Metabolic diseases, n (%)	13 (15.3)	21 (6.1)	
Other, n (%)	1 (1.2)	4 (1.2)	
Kasai			0.454
Yes, n (%)	40 (47.1)	178 (51.6)	
No, n (%)	45 (52.9)	167 (48.4)	
PELD			0.236
<=14, n (%)	23 (27.1)	118 (34.2)	
>14 and <=23, n (%)	32 (37.6)	135 (39.1)	
>23, n (%)	30 (35.3)	92 (26.7)	
D/R ABO compatibility			0.619
Yes, n (%)	74 (87.1)	293 (84.9)	
No, n (%)	11 (12.9)	52 (15.1)	
**Preoperative factors**			
WBC			0.586
Normal, n (%)	33 (38.8)	123 (35.6)	
Abnormal, n (%)	52 (61.2)	222 (64.4)	
Hb			0.353
Normal, n (%)	72 (84.7)	305 (88.4)	
Abnormal, n (%)	13 (15.3)	40 (11.6)	
**Intraoperative factors**			
Operative time (min)			<0.001**^￠^**
Median (IQR)	438.5 (397–497.5)	398 (360–451)	
Anhepatic phase (min)			0.020**^￠^**
Median (IQR)	40 (35–51)	38 (32–45)	
Blood loss (ml)			0.002^†^
<=200	61 (71.8)	297 (86.1)	
>200 and <=400	17 (20.0)	41 (11.9)	
>400	7 (8.2)	7 (2.0)	
Fluid infusion (ml)			0.008**^￠^**
Median (IQR)	1,810 (1,550–2,400)	1,685 (1,450–2,110)	
RBC transfusion (unit)			0.106^**￠**^
Median (IQR)	1 (1–2)	1 (1–2)	

^**￠**^Wilcoxon rank-sum test; ^†^Fisher exact test.

BW, body weight; GRWR, graft to recipient weight ratio; PELD, pediatric end-stage liver disease score; D/R, donor/recipient; WBC, white blood cell; Hb, hemoglobin; IQR, interquartile range; RBC, red blood cell.

**Figure 1:**
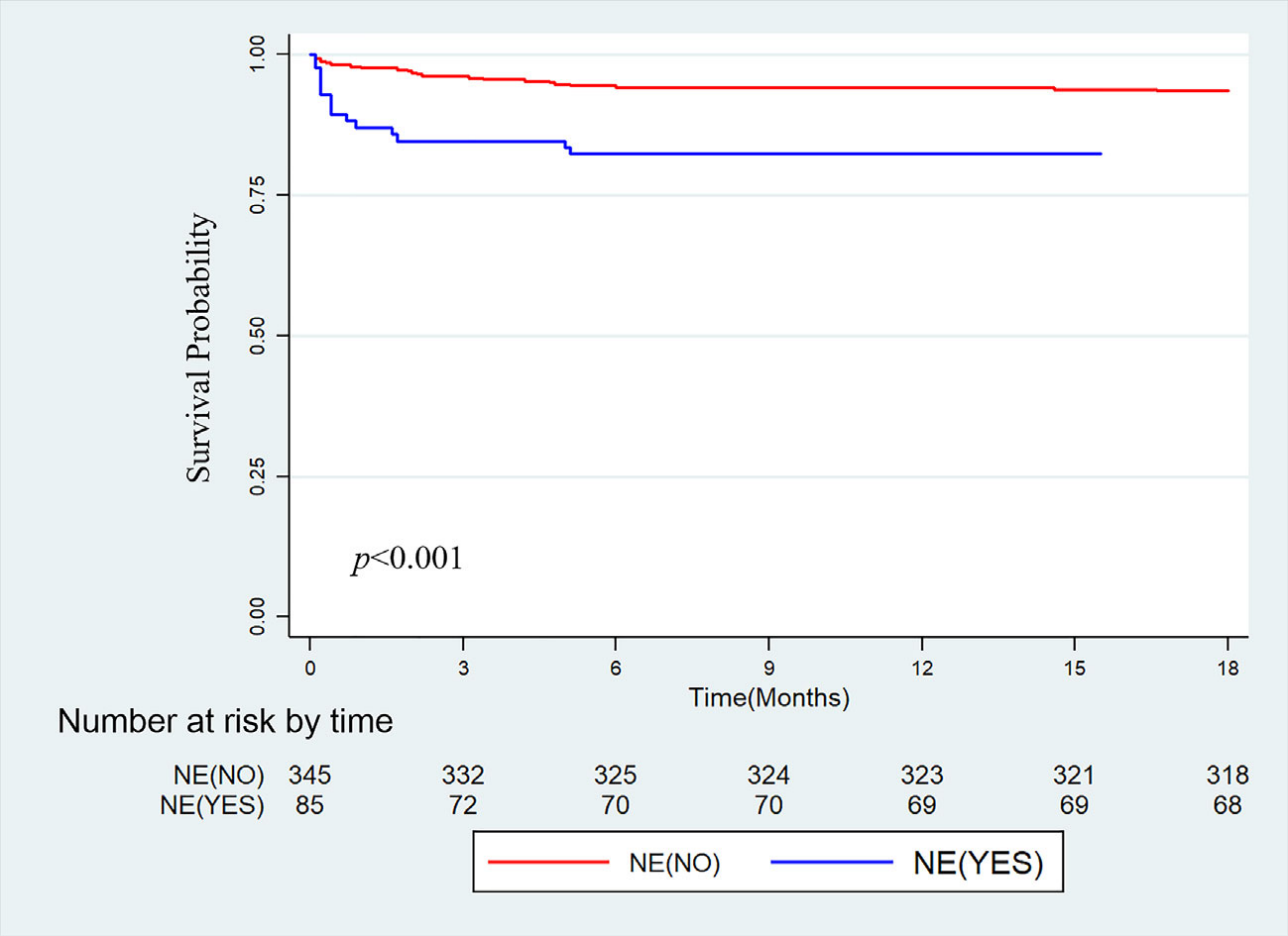
Kaplan-Meier cumulative patient survival for the group without intraoperative norepinephrine infusion (NE-NO) and the group with intraoperative norepinephrine infusion (NE-YES).

The median follow-up time was 658 days (887, 1108) [median (IQR)]. 37 death events happened throughout the follow-up period, among them, 36 (97.3%) pediatric recipients died within 6 months after transplantation and one child survived 439 days before the graft failure. The 1-, 3- and 6-month patient survival rates in non-NE group versus NE group were 97.7% vs. 87.1%, 96.2% vs. 84.7% and 94.2% vs. 82.4%, respectively (log-rank P<0.001; **Figure 1**).

In order to adjust for potential confounding factors, the survival of NE group and non-NE group was compared by multivariate regression and propensity score regression adjustment. Factors with P < 0.1 in the baseline statistical test were then included in the multivariate analysis. The result of multivariate regression analysis suggested that intraoperative NE infusion was an independent risk factor for overall survival after LDLT in pediatric patients, with a hazard ratio (HR) of 2.69 (95% CI, 1.34–5.37), while the other seven variables were not associated with survival according to our results (**Table 2**). In the propensity score regression adjustment analysis, NE was also defined to be associated with survival (p=0.013), with an HR of 2.45 (95% CI, 1.21–4.96) after correction of potential confounding factors (**Table 2**).

**Table 2:** Multivariate regression and propensity score regression adjustment analysis for recipient’s survival.

Variable	HR (95% CI)	P
**Multivariate Regression Analysis**		
NE (yes vs. no)	2.69 (1.34–5.37)	0.005
Age (>24 months vs. <=24 months)	0.80 (0.15–4.36)	0.797
Weight (>10 kg vs. <=10 kg)	1.54 (0.48–4.98)	0.471
Diagnoses Metabolic diseases vs. biliary atresia	0.56 (0.11–2.80)	0.479
Others vs. biliary atresia	NA	NA
Ln (operation-time), min	6.67 (0.71–62.34)	0.096
Ln (anhepatic-phase), min	0.57 (0.15–2.19)	0.415
Blood loss	1.36 (0.70–2.61)	0.364
Ln (fluid infusion), ml	0.86 (0.22–3.38)	0.831
**Propensity Score Regression Adjustment**		
NE (yes vs. no)	2.45 (1.21–4.96)	0.013
Propensity score	19.27 (2.75–135.23)	0.003

NA, not available.

**Figure 2:**
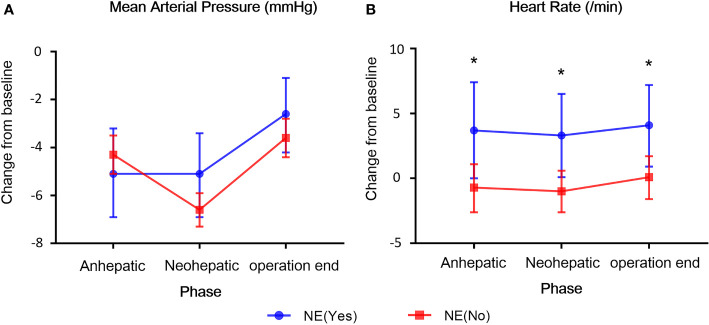
The changes of hemodynamic parameters at the three phases of liver transplantation in both groups. **(A)** Mean arterial pressure (MAP). **(B)** Heart rate. The changes of MAP and heart rate relative to the baseline between the two groups were compared. All the values are presented as mean ± standard deviation. *p < 0.05.

The duration and dosage of NE infusion for patients in NE group were 198 min (108–308) [Median (IQR)] and 138.4 μg (70.4–346.7) [Median (IQR)], respectively. There was no statistically significant difference with regarding to the baseline mean arterial pressure (MAP) (NE group vs. non-NE group, Mean ± SD, 67.3 ± 7.5 vs. 68.6 ± 7.6) and heart rate (NE group vs. non-NE group, Mean ± SD, 114.2 ± 14.3 vs. 117.1 ± 14.4) between the two groups at the beginning phase of the operation (**Table S1**). As the operation proceeded, MAP decreased in both groups, but there was no difference in changes relative to the baseline between the two groups (anhepatic phase, P=0.503; neohepatic phase, P=0.067; operation end phase, P=0.177) (**Figure 2A**). However, compared to the non-NE group, the heart rate of NE group recipients increased more significantly at different phases of operation (anhepatic phase, P=0.025; neohepatic phase, P=0.012; operation end phase, P=0.017) (**Figure 2B**).

## Discussion

Here we reported our experience with 430 pediatric LDLT recipients at a high-volume hospital and demonstrated that intraoperative NE infusion was significantly associated with mortality within 6-month after LT. In this retrospective cohort study, our results have shown that intraoperative NE infusion was associated with poor prognosis for pediatric LDLT recipients, implying that NE might not be the best choice for circulatory support during pediatric LDLT.

In the present study, the change of MAP at each phase of LDLT in NE group was similar to that of patients in non-NE group, while the heart rate of patients in NE group increased significantly at different phases of LDLT operation. These effects of NE on adults’ hearts may be minimal (Ngan Kee et al., 2015; Hasanin et al., 2019), but they can be fatal for pediatric hearts which mainly rely on heart rate to increase cardiac output.

This study highlights the impact of NE infusion on the prognosis of pediatric LDLT. The prevailing opinion seems to be that intraoperative NE infusion was simply a reflection of multiple risk factors such as a sick or septic child, major blood loss, prolonged surgery, reperfusion syndrome etc. However, after eliminating some preoperative and intraoperative confounding factors including preoperative WBC level, PELD score, operative time, the volume of blood loss and fluid infusion by multivariate analysis and propensity score regression adjustment, intraoperative NE infusion persisted as an independent risk factor for recipient survival. Based on our results, we propose that intraoperative NE infusion in pediatric LDLT is reasonable, but not safe. Given that the 1-, 3- and 6-month mortality rates of recipients in NE group versus non-NE group were 12.9% vs. 2.3%, 15.3% vs. 3.8% and 17.6% vs. 5.8%, respectively, clinicians should be fully aware of the potentially adverse consequences when using NE during the perioperative period of pediatric LT.

There are certain limitations in our study. First, this was a single-center retrospective study with potential selection biases including race, surgical techniques, and perioperative management. Second, the preoperative cardiac function of patients was not evaluated because of the insufficiency of cardiac examination data. Third, the low mortality rate was favorable but the statistical analysis was extremely limited. In addition, each anesthesiologist has different tolerance to blood pressure level and different indications for treatment, which leads to great differences in drug use among individuals. Ideally, a multicenter, randomized, prospective study is needed to accurately evaluate the impact of perioperative NE infusion on pediatric LDLT outcomes.

In conclusion, the mortality rate of pediatric LDLT was higher in NE group, suggesting that NE may not be the best choice of vasoconstrictor in pediatric LT. Better preoperative assessment of cardiovascular function and reasonable selection and application of vasoconstrictors will bring benefit to the prognosis of pediatric LDLT. The retrospective characteristic of this study has inherent limitations and prospective analysis would help to reach more convincing conclusions about the impact of NE on pediatric LDLT outcomes.

## Data Availability Statement

The datasets generated for this study are available on request to the corresponding authors.

## Ethics Statement

The studies involving human participants were reviewed and approved by Renji Hospital Ethics Committee, Renji Hospital, Shanghai Jiao Tong University School of Medicine. Written informed consent to participate in this study was provided by the participants’ legal guardian/next of kin.

## Author Contributions

Study design: Y-GL, WZ. Data collection: Y-GL, Z-YP, BQ, and Y-FL. Statistical analysis: WZ and Y-GL. Manuscript drafting: Y-GL, Y-LC, JZ, J-CS, and J-LC. Supervision: W-FY. Project administration: W-FY.

## Funding

This work is supported by the 2019 Development Fund of Anesthesiology, Shanghai Pulmonary Hospital.

## Conflict of Interest

The authors declare that the research was conducted in the absence of any commercial or financial relationships that could be construed as a potential conflict of interest.
