# Supplementary material for: Impact of Intraoperative Norepinephrine Support on Living Donor Liver Transplantation Outcomes: A Retrospective Cohort Study of 430 Children
Source: Front Pharmacol. 2020 Aug 14;11:1254. doi: 10.3389/fphar.2020.01254 (PMC7456957; doi:10.3389/fphar.2020.01254)
Supplement: Supplementary file 1 [file Table_1.docx]

**Table S1**. Hemodynamic parameters at the four phases of LDLT in both groups

| Variables | NE group ( n=85) | Non-NE group ( n=345) |  |
| --- | --- | --- | --- |
| Beginning phase |  |  |  |
| MAP | 67.3 ± 7.5 | 68.6 ± 7.6 | P*=*0.148 |
| HR | 114.2 ± 14.3 | 117.1 ± 14.4 | P=0.099 |
| Anhepatic phase |  |  |  |
| MAP | 62.3 ± 5.6 | 64.2 ± 6.3 |  |
| HR | 118.1 ± 14.7 | 116.3 ± 13.1 |  |
| Neohepatic phase |  |  |  |
| MAP | 62.2 ± 6.2 | 62.0 ± 5.2 |  |
| HR | 117.7 ± 13.1 | 116.0 ± 11.3 |  |
| Ending phase |  |  |  |
| MAP | 64.8 ± 5.4 | 64.9 ± 5.7 |  |
| HR | 118.6 ± 10.8 | 117.1 ± 11.2 |  |

All the values are presented as mean ± standard deviation. MAP, mean arterial pressure (mmHg); HR, hear rate, beats/min.
